# Tuning the Optoelectronic and Photovoltaic Properties of Natural Chlorophyll Dye Molecules via Solvent Interaction: A Computational Insight

**DOI:** 10.3390/nano16060365

**Published:** 2026-03-17

**Authors:** Mohammed A. Al-Seady, Hussein Hakim Abed, Hayder M. Abduljalil, Mousumi Upadhyay Kahaly

**Affiliations:** 1 Department of Theoretical Physics, University of Szeged, Tisza Lajos Krt. 84-86, 6720 Szeged, Hungary; 2 Environmental Research and Studies Center, University of Babylon, Babylon 51001, Iraq; 3 Department of Physics, College of Science, University of Babylon, Babylon 51001, Iraq; hakimhussein.2017@gmail.com (H.H.A.); hayder_abduljalil@yahoo.com (H.M.A.); 4 ELI-ALPS, ELI-HU Non-Profit Ltd., Wolfgang Sandner Utca 3, 6728 Szeged, Hungary

**Keywords:** chlorophyll dye, sensitizer for dye sensitizer solar cells, free energy of electron injection and regeneration, density function theory, open-circuit voltaic

## Abstract

The chlorophyll molecule is considered a low-cost material, easy to synthesize, and easily extracted from plant leaves. It exhibits high chemical stability, structural flexibility, and high absorbance ability at the visible range of electromagnetic radiation. In this work, the geometrical, electronic, and optical properties of pure, dissolved, and doped chlorophyll (C1) natural organic dye were computed by density functional theory (DFT) and time-dependent density functional theory (TD-DFT). The solvents considered include water (H_2_O), acetone (C_2_H_6_O), dichloromethane (CH_2_Cl_2_), chloroform (CH_3_Cl), and dimethyl-sulfoxide (DMSO) (C_2_H_6_OS). The solar photovoltaic parameters, such as light-harvesting efficiency (LHE), oscillation strength (f), free energy of electron injection (${\Delta G}_{Inj.}$) and regeneration (${\Delta G}_{Reg.}$), open-circuit voltaic (V_OC_), and efficiency (η), were also investigated. The evaluated energy gap slightly shifted from 1.920 eV to 1.980 eV based on the solvent polarity, while the UV-Visible absorption spectrum red-shifted from 422.3 nm to 439.8 nm, improving the overall efficiency up to 21.5% in DMSO solvent. The (LHE) and (${\Delta G}_{Inj.}$) properties regarding Cl molecules improved up to 69.1% and −1.384 eV when dissolved in chloroform and DMSO solvents, respectively. Doping C1 molecule via metal transition atoms such as zinc (Zn), nickel (Ni) and copper (Cu) further modified the optical and photovoltaic performance. Doped C1 molecule via Cu atom shows the best photonic results, including the highest open-circuit voltage (V_oc_) and conversion efficiency (Ƞ), while the Ni-doped C1 dye displays the longest lifetime, 1.699 µs, and the highest electronic coupling constant, 1.975 eV; thus, it has the superior photovoltaic performance. These results demonstrate that both solvents and transition metal atom modification significantly improve C1 performance, making metal-doped C1 a promising low-cost and eco-friendly sensitizer for dye-sensitized solar cells (DSSCs).

## 1. Introduction

The rising cost, rapid depletion, and severe environmental impacts associated with fossil fuels have created an urgent need to develop low-cost, sustainable, and environmentally friendly green energy technologies to meet the growing global energy demand [1,2]. The Earth receives approximately 1.75 × 10^17^ W of solar energy per hour, an amount sufficient to satisfy the world’s annual energy requirements. Consequently, solar energy has emerged as a promising renewable energy source capable of replacing fossil-based energy systems [3,4].

In 1954, Fuller, Chapin, and Pearson introduced the first silicon solar cell. However, due to its high production cost, O’Regan and Grätzel later developed modern dye-sensitized solar cells (DSSCs) as a cost-effective alternative to conventional silicon-based photovoltaic devices. DSSCs offer several advantages, including ease of fabrication, lower material costs, and environmental compatibility [5,6].

A dye-sensitized solar cell (DSSC), also known as a Grätzel cell, typically consists of a porous layer of a wide bandgap semiconductor (such as titanium dioxide, TiO_2_) coated with a light-absorbing natural dye, deposited on a transparent conductive glass electrode, along with an electrolyte layer and a counter electrode often coated with graphite or platinum [7,8].

In recent years, DSSCs have gained substantial attention as a next-generation photovoltaic technology due to their low cost, flexibility, eco-friendliness, and high light-to-electricity conversion efficiency [8,9]. Researchers continue to explore new and more efficient electrolytes, sensitizers, and semiconductor materials to further enhance the performance and durability of DSSCs [9].

Research related to DSSC efficiency is generally the investigation of three essential components: natural dye, semiconductor electrodes, and redox electrolyte reactions [10,11]. Solar cell efficiency depends on the amount of light absorption that can be investigated [12]. Likewise, the electron excitation process on the dye due to solar radiation, electron injection ability from dye to semiconductor, and electron collection capability of the natural dye–semiconductor interface also alter the efficiency of DSSCs [13,14]. The oxidized dye would be able to reproduce electrons, which is highly dependent on the kind of natural dye and the type of electrolyte [15].

In DSSCs, natural dyes can be utilized as a dye sensitizer, which is produced from fruits, leaves, flowers, tree bark, and roots, etc. [16]. Natural dyes have many advantages, being widely available, simple to prepare, non-toxic, and biodegradable [14]. Natural pigments that can be adopted as dye sensitizers involve carotenoids, flavonoids, chlorophyll, and anthocyanin, which can be obtained from plants cheaply [12,13]. The chlorophyll molecule has been commonly investigated as a sensitizer in DSSCs [17]. It exhibits visible absorption characteristics due to the presence of a porphyrin structure containing a conjugated pyrrole system, and it is composed of substituents on the sides. The difference between the HOMO and LUMO energy levels with a small band gap energy, low-cost materials, simple preparation process, high molar absorption coefficient, and excellent chemical and thermal stability make it a perfect sensitizer [17,18,19].

In 2017, Faiz et al. [20] theoretically investigated the effect of various solvents (water, ethanol, hexane, and DMSO) on the electronic and optical properties of natural chlorophyll dye using density functional theory (DFT). They found that the band gap decreased from 1.715 eV to 1.468 eV, indicating that the excitation energy is highly sensitive to the solvent environment. Their results confirmed that chlorophyll is a suitable sensitizer for dye-sensitized solar cells (DSSCs).

In 2018, Qian Liu et al. [21] studied the structural and photoelectrical properties of six natural chlorophyll dyes extracted from different plant leaves for use in dye-sensitized solar cells (DSSCs). Using density functional theory (DFT) and time-dependent DFT (TD-DFT), they analyzed the ground and excited states, HOMO-LUMO energy levels, oxidation potentials, and absorption characteristics of the dyes. The HOMO energies ranged from −4.33 to −4.74 eV, and LUMO energies from −2.46 to −6.43 eV. The onset oxidation potentials varied between –0.07 and 0.34 V. TD-DFT results showed absorption wavelengths in the visible range (405.87–621.44 nm), consistent with experimental absorption peaks at 400–420 nm and 650–700 nm, both with and without TiO_2_ clusters. The conversion efficiencies of the dyes ranged from 0.3% to 1.1%, with the D1 chlorophyll dye exhibiting the highest efficiency (≈1.1%) and a fill factor of about 0.71.

In 2018, Zanjanchi and Beheshtian [22] used DFT and TD-DFT calculations to study 12 natural chlorophyll pigments for dye-sensitized solar cells (DSSCs). They found that all pigments absorbed visible light in the 374–481 nm range, with β-carotene and lycopene showing the highest oscillator strengths (3.834 and 4.414, respectively). Theoretical and experimental absorption spectra were in good agreement. They observed that dissolving dyes in different solvents improved the free energy of electron injection (e.g., chlorophyll-c1 increased from 1.101 to 2.1 in water) and enhanced light-harvesting efficiency (LHE of chlorophyll-c1 rose from 0.61 to 0.89 in acetonitrile). The study concluded that solvent effects significantly enhance the optical properties of natural pigments, supporting their potential use as sensitizers in DSSCs.

In 2022, H. Abed et al. [23] studied the influence of graphene quantum dots on the electronic and optical properties of phthalocyanine and curcumin natural dyes using density functional theory (DFT). They found that pure phthalocyanine and curcumin absorbed in the visible range, with phthalocyanine showing Q and B bands at approximately 610 nm and 450 nm, and curcumin exhibiting an absorption peak around 390 nm. Incorporating graphene shifted the UV-Visible spectra of both dyes towards the red region, indicating enhanced light absorption. The study concluded that the graphene–phthalocyanine nanocomposite is more suitable as a sensitizer for dye-sensitized solar cells (DSSCs) than the graphene–curcumin nanocomposite.

**Figure 1:**
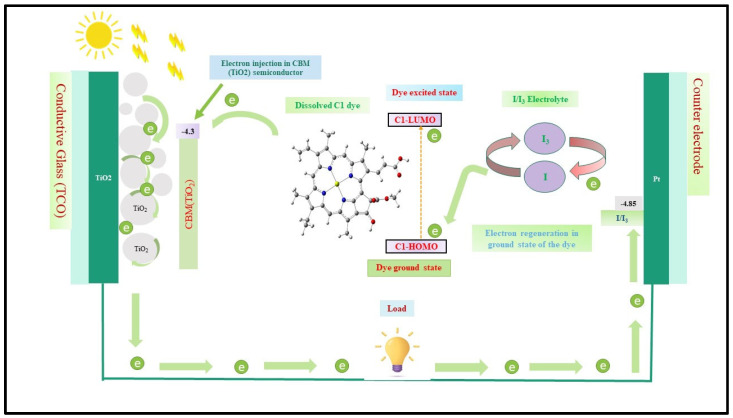
Illustration of the mechanism of electron injection and regeneration in the CBM(TiO_2_) semiconductor and ($I^{-}/I_{3}^{-}$) electrolyte. Based on this mechanism, the sensitizer of the organic dye solar cell will work.

Figure 1 describes the principal operational work of the DSSC system such as electron injection into TiO_2_ semiconductor materials and electron regeneration into the electrolyte. Herein, the sensitizer element is chlorophyll, the electrolyte is iodine/tri-iodine and the collected electrode is titanium-dioxide (TiO_2_). As seen in the figure, the electron injected into the conduction band of the CBM(TiO_2_) and regenerated in the ($I^{-}/I_{3}^{-}$) electrolyte. It clears that electron injection from the excited state of dissolved C1 dye into the CBM(TiO_2_) semiconductor and electrons flow through the circuit to the load (a lamp, for example) until it reaches the $I^{-}/I_{3}^{-}$ electrolyte and the electron regenerates in the ground state of the dissolved C1 dye, and, again, injects into the conduction band of the TiO_2_ semiconductor material.

In the present study, we would like to prepare the overall investigation regarding the dissolved and doped chlorophyll molecule. In addition, we aim to improve the performance of chlorophyll in DSSC application. Because of the interaction between the valence electron belonging to chlorophyll and solvent particles, the photovoltaic parameters will be improved. In the same line, the doped process enhances the molecular properties due to the effect of the metal–coordinate ligand bond between transition metal and nitrogen atoms regarding chlorophyll molecules. In general, the interaction between nitrogen and transition metal forms a fundamental metal–ligand coordination bond resulting from the donation of a lone pair of electrons from nitrogen to the copper center with a partial covalent character. Therefore, the aim of the present study is to investigate the effect of the different chemical solvents and metal transition atoms on the electronic, optical and photovoltaic properties of the chlorophyll molecule and, in addition, to investigate the ability to use chlorophyll as a sensitizer in dye solar cell sensitizers.

## 2. Methodology

### 2.1. Density Function Theory (DFT)

Density functional theory (DFT) is a highly accurate quantum mechanical simulation method widely used to study the electronic, structural, and optical properties of many-body systems. It is based on electron density as the primary variable, allowing molecular properties to be calculated without relying on complex many-body wave functions. DFT provides an effective balance between accuracy and computational efficiency, making it suitable for analyzing large molecules, clusters, and low-dimensional structures. By combining the Hohenberg–Kohn theorems with the Kohn–Sham formalism, DFT enables the computation of a wide range of physical, chemical, and material properties. The central concept of DFT is the electron density (n(r)), which represents the probability of finding an electron at a specific location and can be derived from the many-body wave function through integration over all electron coordinates [24,25,26,27].

(1) $$n\left(r\right)=N\int |\psi (r_{1},\;r_{2},\ldots ,r_{N}{|}^{2}){dr}_{2}{dr}_{3}\ldots {dr}_{N}$$

### 2.2. Computational Methods

The geometrical, electronic, optical and photovoltaic characterization regarding isolated and dissolved chlorophyll molecules (C_33_H_31_N_4_O_5_Mg) are evaluated via density function theory and time-dependent density function theory tools. The exchange-correlation is the most essential tool for describing the electron property and charge transfer. We selected Beck’s three parameters and the Lee–Yang–Parr (B3YLP) functional to describe the exchange-correlation at the level B3LYP/6-31G*. The most popular hybrid functional, B3LYP uses Becke’s 1988 exchange functional ($E_{X}^{B88}$) and Lee, Yang, and Parr’s correlation functional ($E_{C}^{LYP}$) as gradient corrections to the LSDA exchange and correlation functionals. The B3LYP hybrid function can be described by the following formula:

(2) $$E_{XC}^{B3LYP}=\left(1-a\right)E_{X}^{LSDA}+aE_{XC}^{HF}+bE_{X}^{B88}+cE_{C}^{LYP}+\left(1-c\right)E_{C}^{LSDA}$$

where the three parameters are a = 0.20, b = 0.72 and c = 0.81.

The optical properties of the molecules under study, such as the maximum wavelength of absorption, oscillation strength, vertical excitation energy, and light-harvesting efficiency, were carried out by time-dependent density function theory (TD-DFT). In addition, the density of state for the molecule under investigation was computed by GaussSum 3.1 software. The highest occupied molecular orbitals (HOMO) and lowest unoccupied molecular orbitals (LUMO) are useful to understanding the charge transfer between the conduction band of TiO_2_, and the chlorophyll molecule is also investigated. In the present study, we will utilize five types of solvents for dissolving the chlorophyll molecule: water (H_2_O), chloromethane (CH_3_Cl), dichloromethane (CH_2_Cl_2_), acetone (C_2_H_6_O) and dimethyl-sulfoxide (C_2_H_6_OS). All calculations are evaluated by Gaussian09 software; in addition, the two-dimension structures are generated via nanotubes modular. Gaussian 5.0 software is utilized for generating the solvent environment by activating the solvent mode (model = default). The optimum constants used during the DFT calculations are temperature (300 °K) and pressure (1 atm), in addition to a self-consistent filed (SCF) convergence criterion of $10^{-6}$ eV.

### 2.3. Theoretical Approach

The efficiency conversion from photon-electricity (η) can be deduced by the short-circuit current ($J_{SC}$) and open-circuit voltaic (V_OC_). The efficiency can be written as follows [28]:

(3) $$\eta =FF\times \frac{Jsc\;Voc}{P(inc)}$$

where FF is the filling factor and P(inc) is the incident light power on the cell. The stander value of P(inc) at 100 mW/cm^2^ and 1.5 AM is 15 mA/cm^2^. The short-circuit current density ($J_{SC}$) can be described as follows [29]:

(4) $$J_{SC}\;=\;\int_{\lambda }\;LHE\left(\lambda \right)\emptyset_{inject}\;{Ƞ}_{collect}\;d\lambda$$

The filling factor concept helps in computing the efficiency of the solar sensitizer device; in addition, a higher filling factor property gives us a higher efficiency of conversion. In the same line, it computes based on the open-circuit voltaic property. Formula (3) explains the empirical mathematical expression related to FF property.

(5) $$FF=V_{OC}-\frac{\ln\left(V_{OC}+0.72\right)}{V_{OC\;}+1}$$

LHE(λ) is the light-harvesting efficiency, $\emptyset_{inject}$ is the electron injection efficiency and ${Ƞ}_{collect}$ is the efficiency of charge collection. To discuss $\emptyset_{inject}$ and LHE in order to deduce high $J_{SC}$, the efficient dye sensitizer used for DSSC has high LHE and can be described as the following relation [24,25]:

(6) $$LHE=1-10^{-f}\;\;\;\;\;\;\;\;\;$$

where (*f*) is the oscillation strength of the dye corresponding to the maximum wavelength of absorption ($\lambda_{Max}$). The value of the electron injection property is affected by the low open-circuit voltaic (V_OC_) and the short-circuit current ($J_{sc}$) in DSSC. Generally, the free energy of electron injection can be determined by the relax and unrelax path; the present paper will use the unrelax path reliable. From Relation (3), there is a probability of deducing a high value of Jsc, with high LHE, *f* and $\emptyset_{inject}$ corresponding to the driving force of the electron injection (${\Delta G}_{Inj.}$) from an excited state of dye to a minimum conduction band of titanium dioxide ${CBM}_{{(TiO}_{2})}$ anatase phase, which can be evaluated by the unrelax path as follows [26,27]:

(7) $${\Delta G}_{Inj.}=\;E_{ox}^{dye*}-E_{CB}\left({TiO}_{2}\right)$$

where $E_{ox}^{dye*}$ is the energy of oxidation potential of stationary dye, and $E_{CB}\left({TiO}_{2}\right)$ is the minimum conduction band value of the TiO_2_ semiconductor (−4.30 eV).

The $E_{ox}^{dye*}$ can be deduced from the following relation [30,31]:

(8) $$E_{ox}^{dye*}=E_{ox}^{dye}-E_{\left(\lambda max\right)}$$

where $E_{ox}^{dye}$ is the ground state of the redox potential ($I^{-}/I_{3}^{-}$), and $E_{\left(\lambda_{Max}\right)}$ is the excitation energy corresponding to a maximum wavelength of absorption [32].

The regeneration energy for the dye can be computed from the following equation:

(9) $${\Delta G}_{Reg.}=\;E_{ox}^{dye}-E_{redox}^{electrolyte}$$

where the term $E_{redox}^{electrolyte}$ is the redox potential of ($I^{-}/I_{3}^{-}$) electrolyte (−4.85 eV).

According to the relation between the open-voltaic circuit and the dye (E_LUMO_), based on injection, the electron E_LUMO_ → CBM(TiO_2_) is given by [33,34]:

(10) $$V_{OC}=E_{LUMO}-E_{CB}\left({TiO}_{2}\right)$$

## 3. Results and Discussions

### 3.1. Relaxation Structure and Total Energy

**Figure 2:**
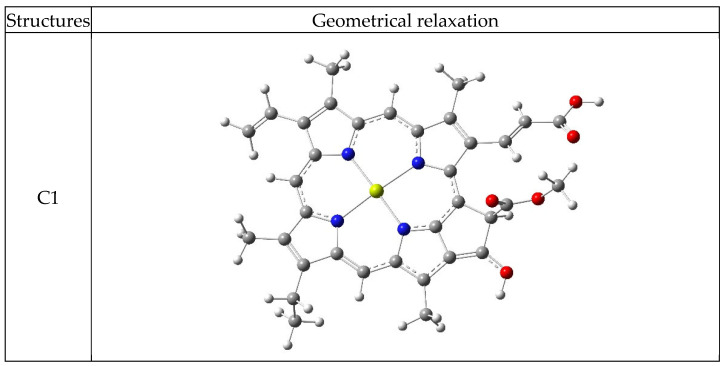
Illustration of the full geometrical relaxation structure regarding the chlorophyll dye. The white, green, red, gray and shallow yellow are hydrogen, nitrogen, oxygen, carbon, and magnesium, respectively.

Figure 2 lists the geometrical structure of isolated chlorophyll dissolved in the water and dimethyl-sulfoxide (DMSO) that was analyzed by the DFT simulation method. The parameter of the geometrical relaxation in a ground state, such as bond lengths and bond angles, was computed. The DFT results showed that the different types of solvents were unaffected by the nature of the bond length and bond angle of the chlorophyll. In addition, the distribution of molecular orbitals was similar in the pure and dissolved state.

DFT calculation deduces that the total energy of the chlorophyll molecule is affected by the type of solvent, and the structure becomes more stable, especially when the chlorophyll is dissolved in water and DMSO solvents. Table 1 listed the computed values of total energy and geometrical parameters for chlorophyll molecules in the pure and dissolved state (water and DMSO). Our results agreed with a previous study [35,36,37,38].

**Figure 3:**
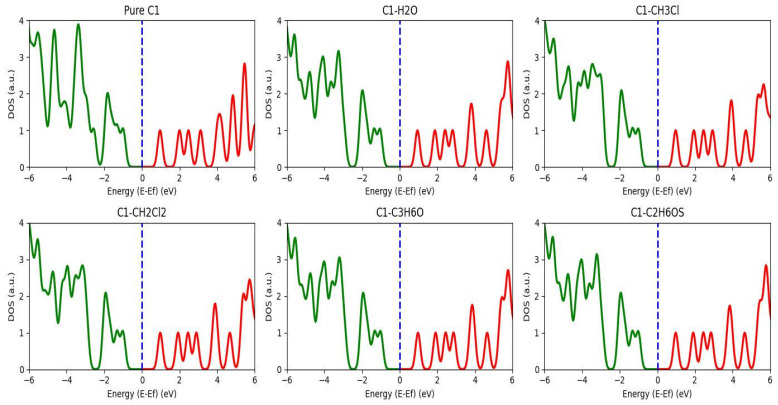
Illustration of the density of state for pure and dissolved chlorophyll natural organic dye in solvents (CH_3_Cl, CH_2_Cl_2_, CH_3_H_6_O, C_2_H_6_OS, and H_2_O), respectively.

Figure 3 lists the density of state (DOS) spectrum for pure and dissolved chlorophyll molecules. The figure deduced that the energy of the molecular orbitals for dissolved chlorophyll shifted to a higher energy level; for example, the HOMO energy level for isolated chlorophyll molecule changed from −8.091 to −5.719 eV when dissolved in the H_2_O solvent [25]. The density of state spectrum deduced that the molecular orbital energy of the chlorophyll molecule is affected by the different types of the solvent; likewise, H_2_O and C_2_H_6_OS have a strong effect on the density of state spectrum of the chlorophyll natural organic dye compared with other solvents. In addition, the different types of solvent affect the energy gap, and the result indicated that the energy gap becomes wide due to the effect of the solvents; for example, when the chlorophyll is dissolved in the water, the energy gap shifted from 1.959 eV to 1.991 eV. In general, we can explain the effect of the chemical solvents on the energy gap property regarding the C1 molecule. The solvent itself produces tiny particles because of the nucleation process. These particles are in nano-meter range; thus, molecules or semiconductor materials can generate a quantum confinement impact. During the confinement process, the mobility of electrons and holes is limited regarding the solvent particle size. This is why the energy needed to excite the electron from the valence band to conduction band increases.

### 3.2. Electronic Properties for Dissolved C1 Natural Dye

In the following part, the DFT method is utilized to calculate molecular orbital energy levels and band gap energy for chlorophyll (types C1) in the isolated and dissolved cases; in addition, the free energy of electron injection and regeneration, based on E_HOMO_ and E_LUMO_ criteria, is evaluated. One of the most essential parameters to determine the performance of the DSSC is the molecular orbital energy level distribution around the ${CBM}_{{(TiO}_{2})}$ and $I^{-}/I_{3}^{-}$ electrolyte [35,36,37]. The important condition for continuous electron injection in the conduction band of the TiO_2_ semiconductor is that all LUMO levels populate above the ${CBM}_{{(TiO}_{2})}$ and HOMO levels must be distributed below $I^{-}/I_{3}^{-}$ [39,40]. Table 2 lists the values of molecular orbital energy and energy gaps for pure and dissolved chlorophyll natural organic dye.

**Figure 4:**
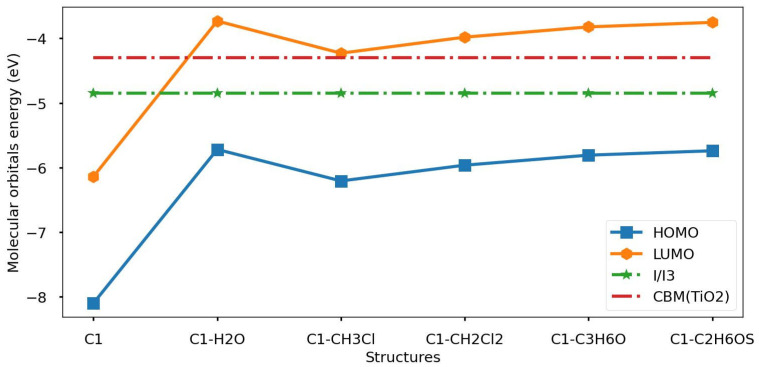
Visual of the population of the molecular orbital energy level for pure and dissolved chlorophyll dyes around the ${CBM}_{{(TiO}_{2})}$ semiconductor and ($I^{-}/I_{3}^{-}$) electrolyte.

From the results, the HOMO energy level localizes below the redox potential of $I^{-}/I_{3}^{-}$ electrolyte, which is why C1 can generate electrons at the ground state. In the same line, the LUMO energy level of the C1 molecule localizes below the ${CBM}_{{(TiO}_{2})}$, so its ability to inject electrons from an excited state to the ${CBM}_{{(TiO}_{2})}$ is insufficient. Therefore, we want to improve the electron injection ability of C1 molecules by using polar chemical solvents. To do so, we select different solvents varying in dissolvability. The chemical solvents have a direct impact on the electronic properties of C1 molecules, so we can easily observe the modification process. In addition, the photovoltaic properties related to C1 molecules will be enhanced. As shown result in table, LUMO energy levels of C1 molecules improved and shifted to a higher energy range. Furthermore, LUMO energy levels distributed above ${CBM}_{{(TiO}_{2})}$ semiconductor materials. Thus, the ability of the C1 molecule to inject electrons into ${CBM}_{{(TiO}_{2})}$ is improved because of the impact of chemical solvents. Meanwhile, all HOMO levels are localized below the $I^{-}/I_{3}^{-}$ electrolyte, which means that the electron can transfer to the ground state of the chlorophyll molecule; thus, the injection and regeneration processes will occur [41,42]. It is worth noting here that future experimental studies at high-repetition-rate facilities such as ELI-ALPS could directly probe solvent- and metal-dependent electron injection and relaxation dynamics in doped chlorophyll systems, thereby bridging the present computational findings with ultrafast photophysical measurements relevant to device optimization [43]. Figure 4 illustrates the distribution of the molecular orbital energy levels of isolated and dissolved chlorophyll around the ${CBM}_{{(TiO}_{2})}$ and $I^{-}/I_{3}^{-}$ measured in electron volt units (eV).

### 3.3. Photovoltaic Parameters

Based on molecular orbital energy level (HOMO and LUMO) conditions, we can determine whether any molecular system is acceptable for solar sensitizers of DSSCs. As photonic devices or solar sensitizer in DSSC applications, the HOMO/LUMO levels must satisfy the requirements of the electrode and electrolyte for transporting electrons from the electrolyte to the LUMO energy level of molecular systems/natural dyes (sensitizers). Upon photoexcitation, an electron is promoted from the HOMO to the LUMO level of the sensitizer and subsequently injected into the CBM of TiO_2_. The oxidized dye is then regenerated by electron donation from the electrolyte, thus completing the ideal electron injection–regeneration cycle inside the sensitizer. The key photovoltaic parameters governing this process include the free energies of electron injection ${\Delta G}_{Inj.}$ and regeneration ${\Delta G}_{Reg.}$, light-harvesting efficiency, fill factor, and the overall power conversion efficiency [44,45].

To gain a deeper understanding of the electron injection and regeneration processes, we consider an ideal photovoltaic cycle that begins with photoinduced electron transfer from the HOMO to the LUMO level of the sensitizer. This excitation is followed by two essential steps: (i) electron injection from the excited LUMO into the ${CBM}_{{(TiO}_{2})}$ semiconductor, and (ii) regeneration of the oxidized dye by electrons supplied from the redox electrolyte. In addition, the potential difference between the redox potential of the electrolyte and the oxidation potential of the dye act as the main driving forces for efficient dye regeneration. A sufficiently large potential gradient not only enhances the regeneration rate of the dye but also suppresses interfacial charge recombination between the oxidized dye and electrons in the ${CBM}_{{(TiO}_{2})}$, thereby improving the overall photovoltaic performance of the DSSC [46].

Table 3 lists the photovoltaic parameters for isolated and dissolved chlorophyll organic dye. Firstly, we compute the free energy of electron injection and regeneration for the C1 molecule into isolated and dissolved cases. The result indicated that isolated chlorophyll dye has a positive range of ${\Delta G}_{Inj.}$, so the pure chlorophyll cannot inject the electron into ${CBM}_{{(TiO}_{2})}$; in addition, the LUMO energy level localizes below ${CBM}_{{(TiO}_{2})}$ semiconductor materials. The free energy of electron injection is essential in the process of electron injection, which is why we dissolve the C1 molecule into different types of chemical solvents. For all dissolved chlorophyll dyes, ΔG shifts into a negative energy range due to the interaction between the C1 molecule and solvent particles. As a result, we deduced that the dye excited state located above the ${CBM}_{{(TiO}_{2})}$ and dissolved chlorophyll dye will normally inject the electron from the excited state of the dissolved chlorophyll to ${CBM}_{{(TiO}_{2})}$ [47]. In addition, the computed values of ${\Delta G}_{Reg.}$ are in a negative range for both phases (isolated and dissolved phases). Results show that ${\Delta G}_{Reg.}$ shifts towards a higher energy range, which is why the probability of electron regeneration into the ground state increases. Finally, the high value of free energy of electron injection ${\Delta G}_{Inj.}$ and low value of ${\Delta G}_{Reg.}$ will produce a faster charge transport between the chlorophyll dye and electrolyte [48]. It is worth noting that high values of ${\Delta G}_{Reg.}$ energy indicate the efficient electron-regeneration capability of the dissolved C1 natural dye, i.e., favorable energy for transferring the injected electron back to the ground state of the dye molecule. This directly contributes to enhancing the short-circuit current density ($J_{SC}$) of the DSSCs, since faster and more favorable dye regeneration suppresses recombination pathways and maintains continuous electron injection. Moreover, the order of the ${\Delta G}_{Reg.}$ for the dissolved C1 dye in the investigated solvents follows the trend C1-CH_3_Cl > C-CH_2_Cl_2_ > C1-C_3_H_6_O > C1-C_2_H_6_OS > C1-H_2_O. This sequence reflects the influence of solvent polarity and solvation effects on stabilizing the oxidation dye state and modulating the regeneration driving force.

Open-voltaic circuits, or open potential circuits, in the DSSC field illustrate the maximum voltage/potential generated by the cell when no external load is applied. The Voc property is closely related to the band gap energy between the minimum conduction band of semiconductor materials and the redox potential of the electrolyte, and it plays an essential role in the overall efficiency of DSSCs. A theoretical V_OC_ value can be computed via the empirical formula that is explained in Relationship (10). In addition, the type of electrolyte and semiconductor electrode materials affects the Voc regarding the DSSC. Practically, the dye transfers electrons from its LUMO energy level to the conduction band minimum of semiconductor materials. Overall, if the LUMO energy level is high, Voc will indeed be high. Formula (10) is utilized to evaluate the V_OC_ property based on the difference between the LUMO energy level and the conduction band minimum of semiconductor materials [49,50].

The deduced value of the (Voc) for dissolved chlorophyll dye, computed based on Formula (10), ranged from 1.839 V to 0.569 V; these values are sufficient for efficient electron injection. However, we can deduce the lower value of Voc that is obtained due to the lower energy level of E_LUMO_ [51], and conclude from the above result that the open-circuit voltaic is directly proportional with E_LUMO_. Thus, the narrower band gap energy gives the electron injection a high ability, and open-voltaic circuits become more efficient. As a result, the isolated and dissolved phases of C1 molecules show acceptable values for electron injection into the ${CBM}_{{(TiO}_{2})}$ semiconductor electrode. The recent experimental results show that $V_{OC}^{Exp.}$ from dissolved chlorophyll natural dye is 440 mV when dissolved in water [51]. However, from our simulations, the value of Voc is 569 mV.

The Scherber formula describes the mathematical relation between band gap energy and open-circuit voltaic across a wide range of molecular structures or low-dimension nanomaterials such as graphene and boron-nitride, etc. Scherber found there is a linear relation between energy gap and open-circuit voltaic, and a logarithmic relation with the short-current circuit. This is why the open-circuit voltaic parameter is considered an essential part for controlling the efficiency of conversion. As shown in Table 2, the pure and dissolved C1 molecules have an acceptable band gap energy for DSSC application: it varies between 1.95 till 1.98 eV; therefore, these structures have the ability to harvest photons from sunlight [52].

FF and η parameters are considered an essential factor to determine the efficiency of the sensitizer device. We can evaluate these two properties according to Formulas (3) and (5). In the isolated phase, the efficiency of C1 organic dyes is about 27.2%, but, because the electron injection process is absent, we use chemical solvents for improving electron injection, then the efficiency of the C1 natural organic dye. As a result, shown in Table 3, the efficiency of the C1 dye is improved, especially when dissolved into H_2_O solvent; it is around 7.005% compared with other phases. The ordering of computed efficiency is following (from larger to lower) C1 > C1-H_2_O > C1-CH_2_Cl_2_ > C1-CH_3_Cl > C1-C_2_H_6_OS > C1-C_3_H_6_O. In the case under analysis, the η parameter depends on the Jsc, Voc and FF (direct proportional case), as well as, reversely, P(Inc.). Any increasing and decreasing on $J_{SC}$ directly affects the efficiency of conversion; thus, a high value of $J_{SC}$ gives acceptable efficiency of conversion. In the following study, the $J_{SC}$ value is around (15 mA/cm^2^). As a reminder, despite the C natural dye having a high efficiency of conversion, unfortunately, it cannot inject electrons inside the ${CBM}_{{(TiO}_{2})}$ semiconductor.

## 4. UV-Visible Spectrum

**Figure 5:**
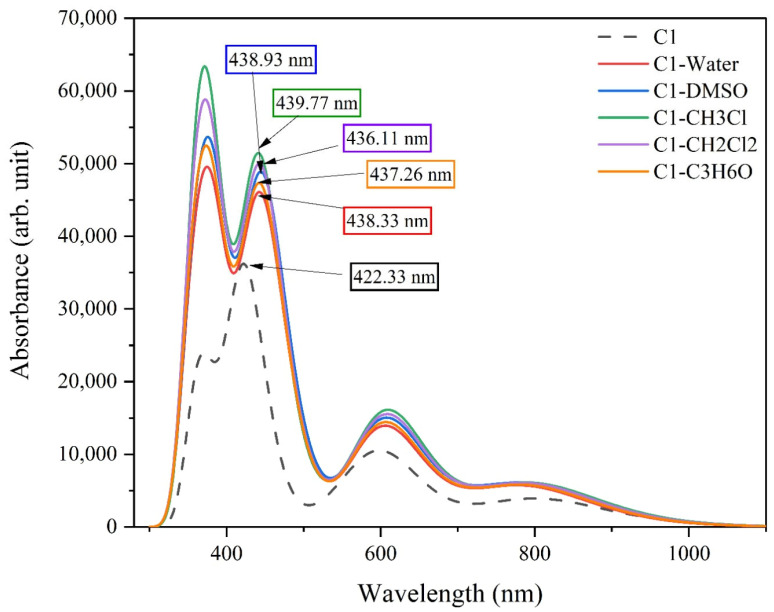
Illustration of the UV-Visible spectra for pure and dissolved chlorophyll dye.

The maximum wavelength of absorption, light-harvesting efficiency, and oscillation strength of pure and dissolved chlorophyll dye were determined by the time-dependent-density functional theory (TD-DFT) method. The simulated absorption spectrum of pure and dissolved chlorophyll dye is presented in Figure 5, and the corresponding maximum wavelength of absorption, electron transition configuration, and oscillation strength are presented in Table 3. Moreover, we investigate the impact of the polar and non-polar solvents in the UV-Visible spectrum regarding the dissolved C1 molecule. We select three polar solvents, for instance, DMSO, H_2_O and C_2_H_6_O, in addition to two non-polar solvents, such as CH_2_Cl_2_ and CH_3_Cl. C1 has two absorption bands in the electromagnetic radiation spectrum: one in the blue-violet range is called the Sort or B-band; the second one absorbs in the red-infrared region called the Q band. Both B and Q bands are found from $\pi \to \pi^{*}$ transitions, where the excited state is described by the notation (π*). The results conclude that pure chlorophyll dye has two transition bands, the first excited state corresponding to a maximum absorption peak of 422 nm, with an oscillation strength of 0.44, which is mainly formed by the electron transition HOMO − 1→LUMO + 1. Meanwhile, the second maximum absorption peak is at 611.25 nm, with an oscillation strength of 0.084, which is mainly generated from the HOMO → LUMO transition. The B3LYP/6-31G* level of theory shows that the theoretical calculation agrees with previous experimental studies [52,53]. We have focused on the B-band to determine the effect of the chemical solvent on the C1 optical properties and another relevant photovoltaic characteristic such as light-harvesting efficiency, free energy of electron injection and regeneration, and efficiency of conversion. Figure 5 shows the computed UV-Visible spectra for pure and dissolved chlorophyll dye. According to the figure, the addition of solvent may increase the shift of the UV-Visible spectrum by several nm in the direction of the red region compared with isolated chlorophyll dye. The change in the position of the absorption peak regarding the dissolved chlorophyll dye is due to the reaction between dye and solvent. For example, the absorption band of dissolved chlorophyll in DMSO and water shifted from 422 nm to 440 nm and 437 nm, respectively. Moreover, the polarity of the solvent is an important parameter that caused the change in the absorption peak of chlorophyll dye. For instance, because of the reaction between polar solvents (H_2_O) and the C1 molecule surface, the absorbance spectrum shifts towards the red range of electromagnetic radiation. Consequently, non-polar solvents also have a positive impact on optical properties regarding the dissolved C1 molecule. Overall, polar and non-polar solvents play an essential role in controlling electric and optical properties regarding the C1 molecule or any molecular system [47,48]. The solvatochromic effect is an important aspect for investigating the impact of solvent polarity on the UV-Visible spectrum regarding organic dye [54]. The present study indicates that the high solvent polarity increases the spectrum, shifting towards the red range of electromagnetic radiation.

**Figure 6:**
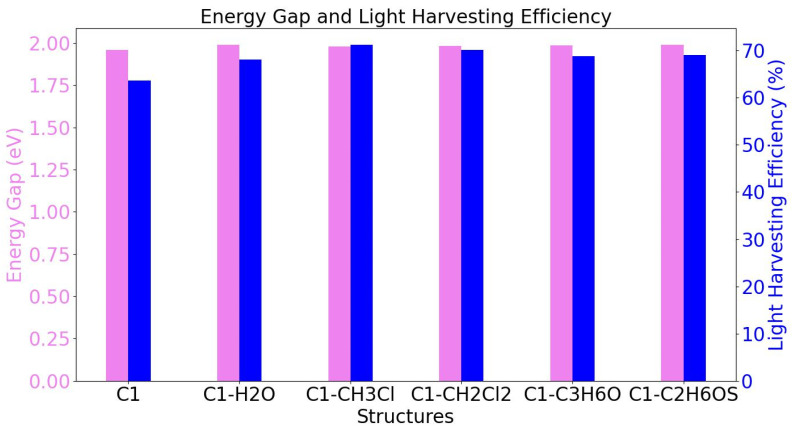
Bar-chart for light-harvesting efficiency and energy gap outcomes with respect to the pure and dissolved chlorophyll dye.

Besides the open-voltaic circuits, maximum wavelength of absorption, and free energy of electron injection, light-harvesting efficiency (LHF) is considered one of the other essential parameters related to the efficiency of DSSCs. Figure 6 illustrates LHF and an energy gap bar-chart describes the performance of the organic dye responsible for the incident light; the LHE is deduced based on Equation (3). The figure displays the bar-cart regarding the LHE for pure and dissolved chlorophyll dye. The computed LHE for pure and dissolved chlorophyll dye varies from 63 to 72%. The highest value of LHE 72% is observed when dissolving the chlorophyll in the chloroform solvent (C1-Chloroform). In parallel, the different types of solvent affect the energy gap, and the result indicated that the energy gap becomes wide due to the effect of the solvents; for example, when the chlorophyll was dissolved in the water, the energy gap shifted from 1.959 eV to 1.991 eV. The doping-induced modulation of the HOMO–LUMO gap and charge-transfer characteristics observed in the present chlorophyll systems parallel the mechanism responsible for the p-type to n-type transition in intercalated graphene structures, highlighting how deliberate atomic-scale modification enables systematic electronic-structure engineering and the controlled tuning of photoresponsivity behavior [55,56]. Based on Figure 6, there is a direct proportional relation between the energy gap and LHE properties, which means any small change in the energy gap property enhances the C1 molecule for harvesting light from sunlight.

## 5. FT-IR Spectroscopy

**Figure 7:**
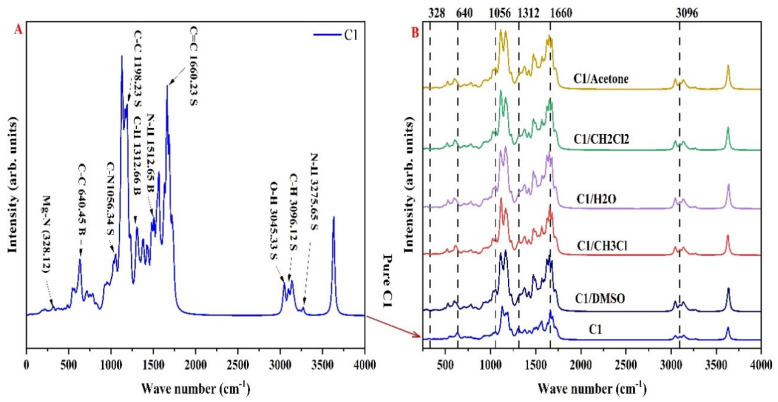
(**A**) Illustration of the FT-IR spectrum for the pure C1 dye measured by the DFT method, the letter S and B referring to stretching and bending vibration modes. (**B**) Illustration of the FT-IR spectrum for the dissolved C1 dye in the different solvents measured via the DFT method.

In the present section, we will investigate the vibrational properties for isolated C1 dye and find the absorption peak related to active groups like C−C,C=C,Mg−N, etc. Then, the C1 dye is dissolved in the different solvents to study the effect of these solutions on the vibration frequency. The DFT method was utilized to find the vibration frequencies of the C1 dye at the level B3LYP/6-31G*. Figure 7A showed the FT-IR spectrum for the isolated C1 dye calculated via DFT at the ground state.

As seen in the figure, the C−C absorption peak is observed at 640.45 and 1198.23 cm^−1^ in bending and stretching modes [54]; furthermore, the C=C group is found at an absorption peak around 1660.23 cm^−1^ [57,58]. The fundamental absorption peak of the Mg-N group is recognized at a vibration frequency around 328.12 cm^−1^. The C−H active group in the bending and stretching modes is noticed at absorption peaks around 1312.66 and 3096.12 cm^−1^, respectively [56]. In addition, the O−H vibration group is detected at an absorption peak around 3045.33 cm^−1^ (stretching mode). The cyanide group (C−N) showed an absorption peak around 1056.34 cm^−1^ [59] (stretching mode). Finally, the N−H group is noticed at absorption peaks around 1512.65 and 3275.65 cm^−1^ [60], and the mode of vibration is bending and stretching, subsequently.

Figure 7B shows the FT-IR spectroscopy for dissolved C1 dye in the different solvents. From the figure, it can be concluded that a small shift occurred in the Mg-N group, around (+32) cm^−1^. Meanwhile, the C−C active group shifted to a lower absorption band, around (22–40) cm^−1^; for example, when the C1 dye was dissolved in the acetone, the absorption peak shifted to 600 cm^−1^. Furthermore, interactions between the C1 dye and solvent particles shifted the C-N group, with an average shift of around (10) cm^−1^. Meanwhile, the C-H absorption peak had an average shift of around (8) cm^−1^. The C=C active group shifted to a lower absorption peak; for example, when the C1 dye was dissolved in the DMSO solvent, the absorption peak shifted from 1660 to 1642 cm^−1^, with an average shift of around (−18) cm^−1^. Finally, the C-H group in stretching mode had a small shift, around (−8) cm^−1^, and it is clear when the C1 dye is dissolved in the CH_3_Cl solvent. From all above results, it can be deduced that the types of selected solvent have a special effect on the absorption peaks and vibration frequencies of C1 dye. For example, active groups such as C−H,Mg−N,C=C and C−N are proportionally affected when the C1 dye is dissolved in the CH_3_Cl and acetone solvents.

## 6. Chlorophyll Metal Derivatives

C1 is considered to be a class from the porphyrin molecular group, and these molecules have application in various prospectives such as charge separation and molecular electronic devices. In addition, CI is widely used as a photo-chemical sensor and in UV-Visible optical devices. In spite of this, it contains many derivatives, and there are only a few works investigating the impact of transition metal on the electronic and optical properties of porphyrin molecules. In addition, porphyrin molecules have a high response to transition metal atoms such as nickel (Ni), cobalt (Co), iron (Fe), and cupper (Cu), etc. This is why we want to check the effect of transition metal on the electronic, optical and photovoltaic characterization of the C1 molecule. The chemical formulas regarding C1-Zn, C1-Ni and C1-Cu are (C_33_H_31_N_4_O_5_Zn), (C_33_H_31_N_4_O_5_Ni), and (C_33_H_31_N_4_O_5_Cu), respectively.

### 6.1. Electronic Properties of C1 Doped via Transtion Metal-Atoms

In the following part, we will investigate the impact doped metal atoms have on the electronic characterization of the chlorophyll molecule and its derivatives, such as molecular orbital energy and band gap property. Table 4 lists the molecular orbital energy and electron injection/regeneration condition band gap property regarding the chlorophyll natural derivatives under investigation.

**Figure 8:**
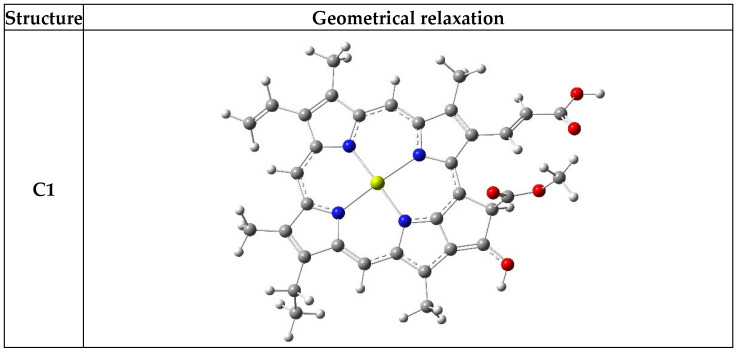

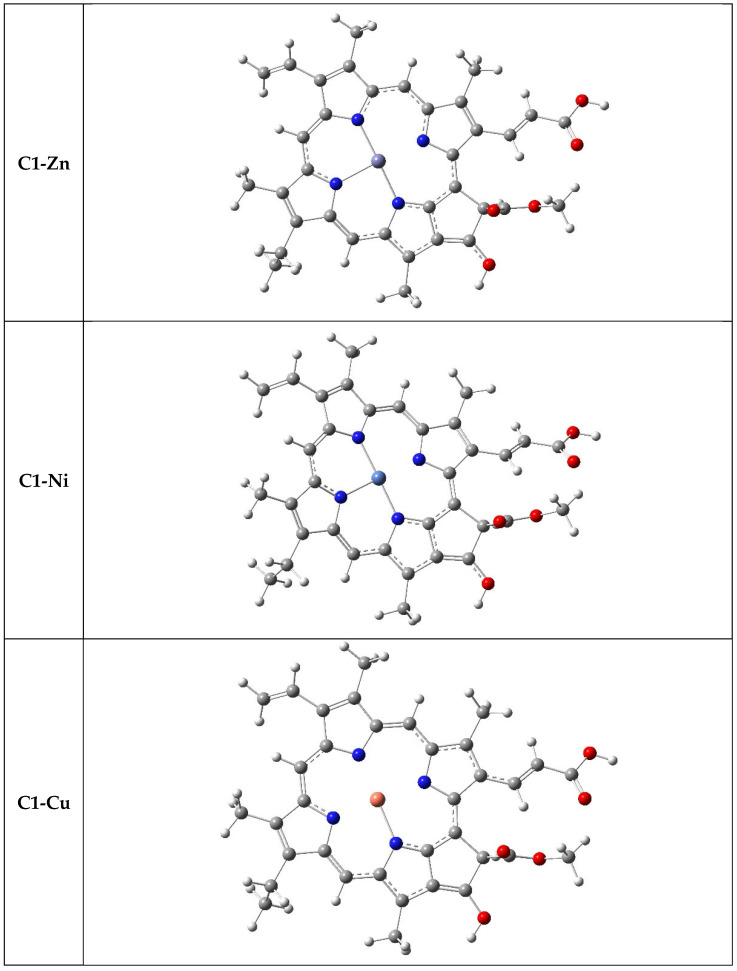
Illustration of the geometrical relaxation regarding the C1 molecule and its derivatives. The white, green, red, gray, light violet, light blue, and shallow pink are hydrogen, nitrogen, oxygen, carbon, zinc, nickel, and copper, respectively.

**Figure 9:**
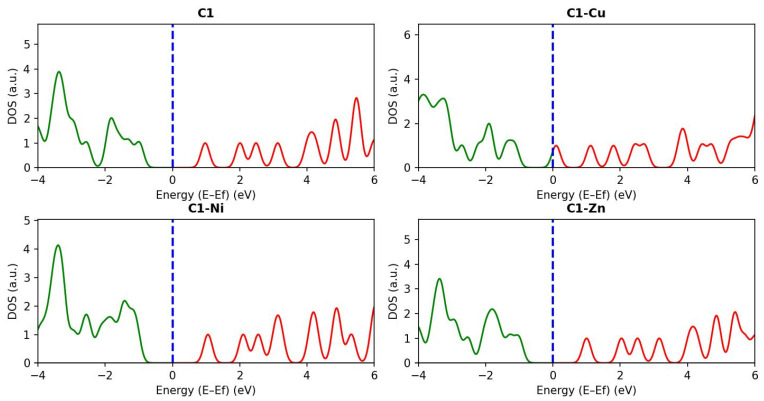
The density of the state spectrum with respect to the chlorophyll molecule and its derivatives. The blue dashed dot represents the fermi level (E*_f_*). The green and red color line represent the range of the HOMO and LUMO energy levels.

As shown, Cu atoms have an extremely high impact on molecular orbital energy and band gap property regarding C1 molecules. It is clear that both HOMO and LUMO energy levels are attributable to C1 molecule shifts to a high energy range, resulting from the impact of the Cu transition metal. Moreover, the band gap of cupper-doped chlorophyll becomes wider than a pristine C1 molecule. Furthermore, the effect of Ni and Zn transition metal atoms must be taken into consideration. The results show that the impact of the Ni and Zn transition metal atoms is smaller than Cu. For instance, the HOMO energy level of the Ni-doped C1 molecule is −8.0164 eV compared with the HOMO energy level of the pristine C1 (−8.099). Thus, we can say, the Cu transition metal has an observable impact on the electronic properties of C1 molecule, higher than Zn and Ni atoms. Figure 8 and Figure 9 show geometrical relaxation and the density of state spectrum regarding the C1 molecule and its derivatives.

### 6.2. Photovoltaic Properties of C1 and Its Derivatives

In the following section, we aim to compute the photovoltaic parameters of C1 dye and its derivatives to check whether these structures are suitable as photosensitizer devices to DSSCs or not. There are many different parameters that can evaluate the performance of photosensitizer devices, such as free energy of electron injection and regeneration, light-harvesting efficiency, open-circuit voltaic, efficiency of conversion and filling factor. In addition, HOMO and LUMO energy levels are considered the key to understanding the mechanism of electron injection and regeneration. In parallel, CBM(TiO_2_) and the $I^{-}/I_{3}^{-}$ model are the most traditional setup for selecting the favorite molecules, nanostructures or composites in DSSC applications. As we mentioned, HOMO and LUMO are considered crucial to photosensitizer applications. Therefore, as we mentioned in Section 3.2, HOMO and LUMO belonging to chlorophyll derivatives must satisfy the limitations of electron injection and regeneration. Then, according to the following conditions, we can determine which molecular structure is acceptable as a photosensitizer. Table 5 visualizes the photovoltaic parameters regarding the C1 molecule and its derivatives.

Recall from Table 4 that we must first investigate the distribution of molecular orbital energy with respect to ${CBM}_{{(TiO}_{2})}$ and the $I^{-}/I_{3}^{-}$ model; then, we can deepen the detail. As shown in the results, all HOMO energy levels distribute below the $I^{-}/I_{3}^{-}$ redox potential, which is why the pristine C1 molecule and its derivatives are able to regenerate electrons into the ground state.

In addition, LUMO energy level localization must be taken into account for investigation of the second part of the conditions. The results show that C1, C1-Zn and C1-Ni cannot inject electrons into the electrode as the LUMO level is below the ${CBM}_{{(TiO}_{2})}$ semiconductor. In spite of this, C1-Cu is able to inject electrons inside the semiconductor materials due to the LUMO energy level localizing above ${CBM}_{{(TiO}_{2})}$. Moreover, the C1-Cu molecular structure has a higher ability to inject electrons than other structures because of the higher rate of free energy of electron injection, meaning a faster recombination process occurs inside C1-Cu.

Moreover, the results indicate that present molecular systems have a high ability to harvest light from the source; furthermore, the ordering of the LHE property is C1-Zn > C1 > C1-Cu > C1-Ni, respectively. Despite the C1, C1-Zn and C1-Ni having the ability to harvest light, these structures cannot be used like photosensitizer devices due to the absence of the electron injection property. As a result, the advantage of the doped process is controlled by the optical properties of the C1 molecule.

**Figure 10:**
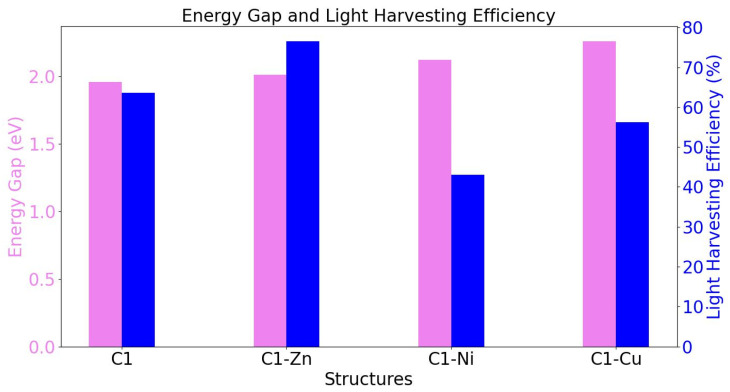
Illustration of the bar-chart of Eg and LHE properties of the pure and doped chlorophyll molecule.

**Figure 11:**
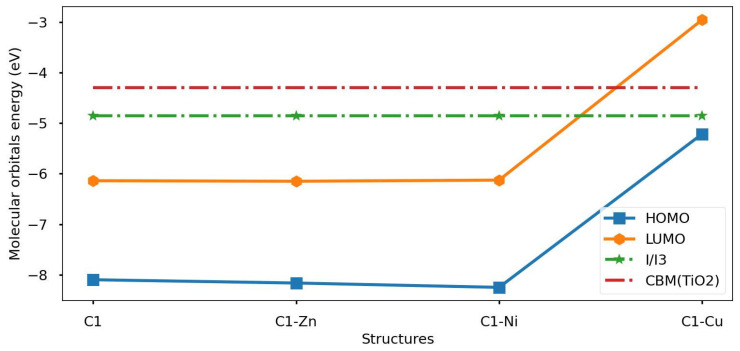
The distribution of molecular orbital energy levels regarding chlorophyll molecule and its derivatives around ${CBM}_{{(TiO}_{2})}$ semiconductor materials and the redox potential of the ($I^{-}/I_{3}^{-}$) electrolyte.

We examined the decreasing/increasing of the LHE property effect on the energy gap property regarding the chlorophyll molecule and its derivatives. The results show that band gap energy regarding chlorophyll derivatives becomes wider than pristine C1 due to the impact of the transition metal atom. Figure 10 shows the bar-chart of Eg and LHE properties regarding the chlorophyll molecule and its derivatives. Finally, the C1-Cu molecular system satisfies all requirements of the photosensitizer devices; it is more acceptable than any other system. Figure 11 shows the molecular orbital population around the minimum conduction band of titanium dioxide and redox potential regarding the iodine/tri-iodine electrolyte.

Another essential factor in solar cell science is efficiency of conversion (ƞ), which describes the amount of sun energy that converts to electric energy. This is why we must evaluate the η property for determining the quality of the molecular system, nanostructures or composites. As shown in the results, the C1 molecule and its derivatives have an acceptable efficiency for converting incident light to electric energy. The order of the η property of C1 and its derivatives follow the arrangement C1-Zn > C1 > C1-Ni > C1-Cu, respectively. Due to the C1-Cu nanostructure satisfying the conditions of electron injection and regeneration, it is more acceptable than any other molecular system, despite it having a lower efficiency than pristine C1 and its derivatives.

As we mentioned in Section 3.3, the Voc is a very important parameter that evaluates the quality of electron injection inside semiconductor electrode materials. In addition, numerous types of factors impact the Voc, such as the work function of the electrode, light intensity, environmental factors and the energy levels of the materials. This is why many theoretical studies show that Voc can be computed via the difference between LUMO energy and the conduction band minimum of titanium-dioxide (−4.3 eV). As shown in Table 5, the Voc values regarding the C1 molecule and its derivatives vary between 1.345 V and 1.839 V. Furthermore, all Voc values with respect to studied molecule structures are in a positive range. The main advantage of positive Voc is that electrons can transfer flex from dye to semiconductor materials. We would like to mention that the experimental calculation depends on the current-voltage (I-V) curve and simulation calculation obtained by the ideal diode limit formula, which is why there are differences between the evaluated outcomes [49,51,61,62].

Overall, simulation investigations show that transition metal atoms improve the photovoltaic parameters regarding the C1 molecule, with computed η in the range of 18–25%. The present values are far higher than the optimum experimental efficiencies, 8–11%, which are evaluated for standard natural organic dyes. Previous reports have recorded the maximum Ƞ with respect to tungsten di-selenium (WSe2) (10.8%) [63], defected graphene (6.40%) [45], and silicon carbide (SiC) (15.03%) [45]. In addition, the Voc regarding the doped C1 molecule is higher than pure graphene (1.089 V) and sulfur-doped nanostructures (0.932 V) [34].

Furthermore, we investigated the effect of the transition metal atoms on the ultra-violet spectrum regarding the C1 molecule and its derivatives. All calculations are extracted via the TD-DFT tool. From the results shown, the maximum wavelength of absorption with respect to the pristine C1 molecule is observed at 422.33 nm; thus, C1 absorbs in the visible range of electromagnetic radiation. Additionally, the outcomes show that, due to the effect of the Ni and Zn transition metals, the UV-Visible spectrum of C1 dye shifts into the blue range (higher energy) of electromagnetic radiation. In contrast, the UV-Visible of the C1 molecule shifts into the red range (lower energy), because of the effect of the Cu atom.

**Figure 12:**
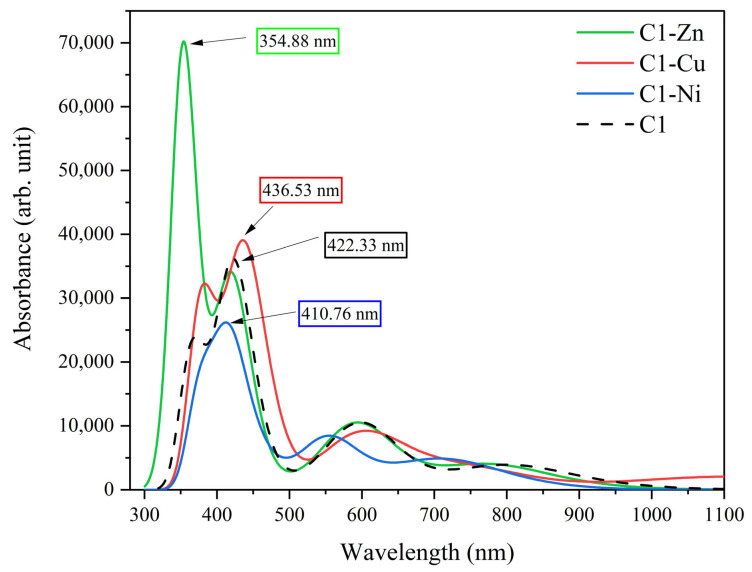
Illustration of the UV-Visible spectrum of the chlorophyll molecule and its derivatives.

Cu atoms play important roles in the UV-Visible spectrum shift. The main reason for the shift is the metal–ligand coordinate that formed between Cu and the N atom inside the C1 molecule center. This is why the electron needs less energy to transfer from the HOMO level to LUMO. Overall, C1 and its derivatives absorbs into the visible range of electromagnetic radiation, but C1-Zn and C1-Ni absorb in the blue range, and C1-Cu absorbs into the red range [64]. Figure 12 shows the UV-Visible spectrum regarding the chlorophyll molecule and its derivatives.

The current study is entirely based on first-principles DFT and TD-DFT simulations. Consequently, all photovoltaic parameters, such as open-circuit voltage (Voc), short-circuit current density ($J_{SC}$), and efficiency, were derived from theoretical relations rather than experimental measurements. It has been reported in the literature, based on experimental results, that the efficiency of DSSCs can indeed be significantly improved by introducing a small composition of ruthenium complex dye into the moss chlorophyll dye [65]. The efficiencies reported in our study thus represent theoretical predictions aimed at providing insight into the effect of solvent polarity on the optoelectronic performance of chlorophyll-based dyes.

Based on Formula (4), there is a direct relation between the short-current density $J_{SC}$ and light-harvesting efficiency; in another form, a higher value of light-harvesting efficiency led to a higher short current. The present study shows that both dissolved and doped chlorophyl natural dye have a high ability to harvest light from the source, resulting in a high potential short-current density and extremely high efficiency of conversion in the visible range. Finally, the proposed chlorophyl molecular structure can be utilized as a solar sensitizer in DSSC devices [61,66].

## 7. Transition Lifetime

The transition lifetime (τ) plays a critical role in determining the photovoltaic performance of DSSCs, as it directly influences the recombination rate and electron injection efficiency. A longer lifetime shows slower recombination of photogenerated electrons with dye molecules or electrolyte species, thereby increasing the probability of successful electron transport into the conduction band of TiO_2_. This behavior enhances the Voc and power conversion efficiency (PCE). The (τ) can be simply evaluated via the numerical formula [67]:

(11) $$\tau =\frac{h\times c}{\Delta E\times f}$$

where h, c, ΔE, and f are plank constant ($h=\;{6.623\times 10}^{-34}\;J\cdot s$), speed of light (${3\times 10}^{8}$ m/s), exaction energy in joule unit, and oscillation strength, respectively. Table 6 illustrates the transition of lifetime with respect to dissolved and doped C1 natural dye.

Furthermore, a longer excited state lifetime allows sufficient time for electron injection from the dye excited state into the semiconductor conduction band, which modifies the short-current circuit density. In contrast, a shorter lifetime increases recombination probability and reduces charge collection efficiency. Therefore, dyes exhibiting longer lifetimes, such as pure C1- and Ni-doped molecular structures, are expected to demonstrate superior photovoltaic performance due to reduced recombination losses and enhanced charge separation.

As shown in Table 6, the evaluated lifetimes values range from 0.557 to 1.699 (μs) for both dissolved and doped C1 dyes. The pure C1 dye shows the longest lifetime in the dissolved state, while Ni doping further increases the lifetime compared to other dopants. This suggests that these systems may exhibit improved charge separation and reduced recombination rates, which are favorable for higher DSSC performance, particularly in terms of Voc and power conversion efficiency. Mathematically, there is a reverse relationship between the lifetime and excitation energy, meaning a higher excitation energy corresponds to a lower transition lifetime, due to the higher interaction rate between the dye electron and incident light. Based on the free energy of electron injection, the results show that both dissolved and doped C1 natural dye have a high ability to inject electrons inside the CBM(TiO_2_); the electron can transfer flex between the excited and ground states. The present study is approximately in agreement with the experimental report in [68], whereby Kosumi et al. have experimentally evaluated the C1 transition lifetime in different solvents. They found that transition lifetime varies between 1.2 and 12 μs.

These findings are consistent with previous experimental and theoretical reports, which confirmed that an extended excited state lifetime improves electron injection efficiency and suppresses recombination processes, leading to modified DSSC efficiency.

## 8. Electronic Coupling Constant

The electronic coupling constant |VRP| plays an essential part in evaluating the electron injection rate from the dye excited state into the conduction band of TiO_2_. According to electron transfer theory, the injection rate is proportional to the square of the electron coupling constant ($k_{inj}\propto {\left|VRP\right|}^{2}$). Therefore, higher |VRP| values deduce faster electron transfer, stronger electronic interaction and more efficient charge separation. Faster electron injections reduce recombination losses and modify charge collection efficiency, which directly enhances short-circuit current density and the overall power conversion efficiency of DSSC devices. Furthermore, stronger electronic coupling facilities better orbital overlap between the dye molecule and TiO_2_ conduction band, resulting in more efficient electron transport across the dye semiconductor interface. The |VRP| can be expressed by the Generalized Mulliken–Hush (GMH) mathematical formula [61]:

(12) $$\left|VRP\right|=\frac{\Delta E_{RP}}{2}$$

(13) $$\Delta E_{RP}=\left[E_{LUMO}^{Dye}+2E_{HOMO}^{Dye}\right]-\left[E_{LUMO}^{Dye}+E_{HOMO}^{Dye}+E_{CBM\left({TiO}_{2}\right)}\right]$$

By enhancing the $\left|VRP\right|$, the performance of DSSC devices will improve, leading to higher electron bonding and more efficient electron transport. Table 7 shows the electronic coupling constant ($\left|VRP\right|$) of the dissolved and doped C1 natural dye, measured by eV unit.

Based on the results, the No-doped C1 dye shows the highest electronic coupling constant (1.975 eV), indicating the strongest electronic interaction and fastest electron injection capability among all studied systems. This suggests that Ni doping significantly modifies charge transfer efficiency and is expected to improve DSSC photovoltaic performance. Conversely, lower |VRP| values observed in some solvent systems indicate weaker electronic interaction and slower electron injection rates, which may reduce the DSSC overall efficiency. The enhanced electronic coupling in the Ni-doped system, due to the strong interaction between Ni d-orbitals and dye molecular orbitals, modifies orbital overlap and facilities faster electron transfer to the TiO_2_ conduction band.

## 9. Conclusions

The effect of solvents and transition metal atoms (Zn, Ni, and Cu) on the optical and photovoltaic properties of the chlorophyll molecule (C1) is investigated. The DFT and TD-DFT computational approaches were employed to analyze the structural, electronic, optical, and photovoltaic characteristics of the pure, solvent-dissolved, and metal-doped chlorophyll systems.

The work revealed that the solvent type has a notable influence on the energy levels and optical behavior of the C1 dye molecule. The impact of the chemical solvent on the geometry, such as bond length and angle between length, is very small. The energy gap slightly increased from 1.920 to 1.982 eV, while the light-harvesting efficiency (LHE) improved, reaching 71.1% in the CH_3_Cl solvent. Solvents also significantly affected the free energies of electron injection and regeneration, with an enhancement to −1.384 eV in DMSO. The UV-Visible spectra showed a red shift in absorption from 422.33 nm to 439.77 nm.

Moreover, doping with transition metals (Zn, Ni, Cu) enhanced the dye’s electronic, optical, and photovoltaic properties, with Cu doping showing the most substantial improvement, making Cu-C1 the most promising sensitizer candidate, while the Ni-doped C1 dye displays the longest lifetime, 1.699 µs, and the highest electronic coupling constant, 1.975 eV; thus, it has the superior photovoltaic performance.

Although the present work deals with theoretical predictions, it is essential to mention that the long-term experimental stability of metal-improved chlorophyll derivatives (Zn-C1, Ni-C1, and Cu-C1) requires further verification, as natural dyes often suffer from photodegradation under real operational conditions. Nevertheless, these metal derivatives remain more environmentally friendly and less toxic than conventional ruthenium-based sensitizers. Future experimental work is needed to evaluate their stability, durability, and practical applicability in DSSCs.

## Figures and Tables

**Table 1:** The relaxation energy, bond length, and bond angle for pure and dissolved chlorophyll natural organic dye in the atomic unit (a.u.), angstrom unit (Å), and degree unit (°), respectively.

Relaxation Energy		Geometrical Parameters			
Structure	Total Energy	Bond Types	Bond Lengths	Angle Types	Bond Angels
C1	−2146.998	C-C	1.468–1.503	C-C-C	113.749–124.693
C1-H_2_O	−2147.015	C=C	1.345–1.379	C-C-H	111.625–111.949
C1-CH_3_Cl	−2146.997	C--C	1.387–1.421	C-C-O	109.589–110.795
C1-CH_2_Cl_2_	−2147.006	C-H	1.083–1.096	C-O-H	107.469–110.701
C1-C_3_H_6_O	−2147.011	O-H	1.355–1.375	C-N--C	106.803–107.562
C1-C_2_H_6_OS	−2147.014	C=O	1.231–1.241	N-Mg-N	176.567–177.731
		C--O	1.339	C-O-H	104.459–110.781
		C-N	1.388–1.420		
		C=N	1.368–1.381		
		Mg-N	2.024–2.106		

**Table 2:** List of the molecular orbital energy and electron injection/regeneration condition regarding pure and dissolved chlorophyll natural organic dye.

Structures	HOMO (eV)	LUMO (eV)	Energy Gap (eV)	Electron Injection and Regeneration Condition
**C1**	−8.099	−6.139	1.96	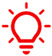
**C1-H_2_O**	−5.719	−3.731	1.988	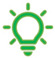
**C1-CH_3_Cl**	−6.207	−4.229	1.978	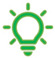
**C1-CH_2_Cl_2_**	−5.963	−3.981	1.982	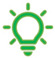
**C1-C_3_H_6_O**	−5.808	−3.822	1.986	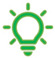
**C1-C_2_H_6_OS**	−5.740	−3.752	1.988	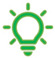

**Table 3:** The free energy of electron injection (${\Delta G}_{Inj.}$), free energy of electron regeneration (${\Delta G}_{Reg.}$), open-voltaic circuit (V_OC_), maximum wavelength of absorption ($\lambda_{Max}$), excitation energy ($E_{ox}^{dye*}$) and oscillation strength (f).

Structure	${\Delta G}_{Inj.}$ (eV)	${\Delta G}_{Reg.}$ (eV)	V_OC_ (V)	LHE	$E_{\left(\lambda_{Max}\right)}$ (eV)	$E_{ox}^{dye*}$ (eV)	f	FF	η (%)
**C1**	0.861	−3.299	1.839	63.6	2.938	5.161	0.441	0.927	22.568
**C1-H_2_O**	−1.411	−0.919	0.569	68	2.831	2.888	0.495	0.821	7.005
**C1-CH_3_Cl**	−0.936	−1.408	0.071	71.1	2.844	3.364	0.54	0.797	5.715
**C1-CH_2_Cl_2_**	−1.174	−1.163	0.319	70.2	2.838	3.128	0.526	0.816	6.692
**C1-C_3_H_6_O**	−1.329	−1.008	0.478	68.5	2.838	2.971	0.502	0.390	0.418
**C1-C_2_H_6_OS**	−1.384	−0.940	0.547	69.7	2.825	2.919	0.519	0.732	3.505

**Table 4:** The molecular orbital energy and energy gap property measured by eV unit.

Structures	HOMO (eV)	LUMO (eV)	Energy Gap (eV)	Electron Injection and Regeneration
**C1**	−8.099	−6.139	1.96	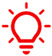
**C1-Zn**	−8.164	−6.149	2.015	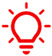
**C1-Ni**	−8.250	−6.127	2.123	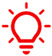
**C1-Cu**	−5.214	−2.954	2.26	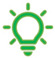

**Table 5:** The photovoltaic parameters of the C1 molecule and its derivatives.

Structures	${\Delta G}_{Inj.}$ (eV)	${\Delta G}_{Reg.}$ (eV)	LHE	FF	VOC (V)	ƞ (%)	λ (nm)
C1	−0.862	−3.299	63.6	0.927	1.839	25.568	422.33
C1-Zn	−0.330	−3.364	76.5	0.928	1.849	25.716	354.88
C1-Ni	−0.971	−3.450	43.1	0.926	1.827	26.391	410.76
C1-Cu	−1.962	−0.414	56.3	0.906	1.345	18.286	436.53

**Table 6:** List of evaluated transition lifetimes with respect to dissolved and doped C1 natural dye, measured in microsecond units.

Structure	τ (μs)
**C1**	0.960
**C1-H_2_O**	0.885
**C1-CH_3_Cl**	0.865
**C1-CH_2_Cl_2_**	0.949
**C1-C_3_H_6_O**	0.809
**C1-C_2_H_6_OS**	0.832
**C1-Zn**	0.557
**C1-Ni**	1.699
**C1-Cu**	1.198

**Table 7:** Illustration of the electronic coupling constant with respect to dissolved and doped C1 natural dye, measured by eV unit.

Structure	|VRP|
**C1**	1.899
**C1-H_2_O**	0.709
**C1-CH_3_Cl**	0.754
**C1-CH_2_Cl_2_**	0.720
**C1-C_3_H_6_O**	0.953
**C1-C_2_H_6_OS**	0.831
**C1-Zn**	1.932
**C1-Ni**	1.975
**C1-Cu**	0.457

## Data Availability

The data presented in this study are available on request from the corresponding author. (These data are going to be part of a PhD thesis, in preparation and not yet public. Due to University privacy policy, the data can be obtained from the corresponding author upon reasonable request.)
